# Quality of Life in Patients Undergoing Cardiac Surgery: Role of Coping Strategies

**DOI:** 10.7759/cureus.16435

**Published:** 2021-07-17

**Authors:** Khizra Iqbal, Yusra Irshad, Syed Rafay Ali Gilani, Shafqat Hussain, Mubashar Ahmad, Usman A Khan, Varda S Choudhry, Aemen S Khakwani

**Affiliations:** 1 Clinical Psychology, Chaudhry Pervaiz Elahi (CPE) Institute of Cardiology, Multan, PAK; 2 Internal Medicine, Kulsoom International Hospital, Islamabad, PAK; 3 Cardiothoracic Surgery, Chaudhry Pervaiz Elahi (CPE) Institute of Cardiology, Multan, PAK; 4 Psychiatry, Chaudhry Pervaiz Elahi (CPE) Institute of Cardiology, Multan, PAK; 5 Internal Medicine and Nephrology, University of Oklahoma Health Sciences Center, Oklahoma City, USA; 6 Internal Medicine, Quaid-e-Azam Medical College, Bahawalpur, PAK; 7 Internal Medicine, Suburban Community Hospital, Norriton East, USA

**Keywords:** coping strategies, quality of life, cardiac surgery, psychological disturbances

## Abstract

Adaptive coping strategies are used to reduce stress in patients undergoing cardiac surgery. These strategies have a major role in physical health, psychological health, quality of life and also affect an individual’s response to the disease. The current study was conducted to comprehend the impact of coping strategies on the quality of life of patients suffering from cardiac disease. A purposive convenient sampling method was used to collect data from different hospitals in South Punjab. We applied Carver's Brief Coping Orientation to Problem Experienced (Brief COPE) inventory and the WHO quality of life scale. A cross-sectional research design was proposed for the study. The findings of the study showed that coping strategies and quality of life are associated with each other, and the use of emotion-focused and problem-focused coping strategies have a significant impact on patients experiencing cardiac surgery. Demographic details of patients also revealed the differences in both variables. Implications and future recommendations have also been discussed.

## Introduction

Cardiovascular diseases (CVDs) have created an alarming situation throughout the globe as they are the leading cause of death and affect 17.7 million (31%) lives per annum. Nonetheless, more deaths are seen due to coronary heart disease in comparison to stroke. Statistics reveal that 7.4 million people die due to the former and 6.7 million from the latter [1]. In Pakistan, the death ratio of CVD patients is 30% to 40%. Unfortunately, the ratio of deaths due to CVDs is 82% in the developing (low-middle income) countries (LMICs) [1]. Cardiac disease is predisposed by genes, gender, age, obesity, poor physical exercise, excessive alcohol intake, insufficient and unhealthy nutrition, predisposition to biology, and a family history of CVD, hypertension (HTN), diabetes mellitus (DM), and hyperlipidemia. Some other non-biological factors like psychosocial stress, lack of food, poor schooling, and air quality [2] lead to psychiatric issues in patients like depression, fear, tension, and anger [3]. These symptoms not only cause unhappiness and mortality but also predict cardiac injury in patients [4] with CVD.

Coping

Coping is defined as the ability to use cognitive and behavioral techniques to reduce stress and psychological distress [5]. In the literature, problem-focused and emotion-focused categories are mostly used as coping strategies [6]. Problem-focused techniques are used to modify cognitive and behavioral responses, such as planning and active coping. In contrast, the emotional response is altered by using emotion-focused techniques such as expressing and positive reappraisal. Problem-focused techniques are more effective as compared to emotion-focused techniques. While dealing with work and family-related issues, individuals employ problem-focused techniques whereas, during the confrontation of health-related and emotional problems, mostly emotional techniques are adapted [7].

Health-related quality of life (HRQL)

It is defined as “the extent to which one’s usual or expected physical, emotional, and social wellbeing is affected by a medical condition or its treatment.” This definition incorporates the two widely accepted aspects of quality of life: subjectivity and multidimensionality [8]. Various researches have also documented an association between the internalization of issues (such as depression, anxiety, tension, and frustration) and inadequate coping strategies [7], thus affecting the quality of life (QOL) of CVD patients indirectly. HRQL is also considered as a differentiating point in assessing disease's wellbeing and decision impact. Biological and lifestyle factors play a significant role in the progression and impact of cardiac disease, which has been pointed out by earlier research [9]. Psychological aspects are often comorbid with physical illness; therefore, it is important to advise management and have prevention measures.

The rationale of the study

Recent research has taken a more balanced course regarding the gender perspective on the quality of life (representation of women and men), concentrating equally on women's and men’s perspectives [10]. In fact, epidemiologic statistics, that is, mortality and life span, are specific and are more favorable to women, so there is a more optimistic discrepancy between the emotional experience, i.e., harmful feelings, and self-reported wellbeing [11]. Relationships are found between life quality and patients' ability to cope with psychological disturbances mediated by cardiac disease [12]. Studies discovered that patients with heart disease who engaged themselves in activities and used problem-solving strategies improved rapidly [13]. Another study also suggests that the use of maladaptive copings strategies lowers the quality of life among patients [14]. In a stressful situation, coping strategies may help patients with cardiovascular disease. As Williams reported, it is important to educate patients regarding stress management by using coping skills [15], and patients who implied these strategies found a significant reduction in psychological disturbances, improving their quality of life.

Cardiac diseases and surgeries are common worldwide, including in Pakistan. Cardiac surgery may be regarded as a traumatic event that leads to emotional distress in patients. This study aims to improve the quality of life of patients with cardiac surgery by using coping strategies so that they can function normally in different walks of life.

Objectives of the study

1. To comprehend the impact of coping strategies on the quality of life of patients who have undergone cardiac surgery.

2. To assess the differences in coping strategies and quality of life on demographic variables.

Hypotheses of the study

1. Coping strategies will be associated with the quality of life among patients.

2. Coping strategies will significantly impact the quality of life among patients with cardiac surgery.

3. Brief Coping Orientation to Problem Experienced (Brief COPE) inventory scale and QOL will differ in terms of demographic variables.

## Materials and methods

Participants

A purposive convenient sampling technique was used to easily approach participants who underwent cardiac surgery at the tertiary care center and were willing to participate in the study. The sample size was calculated by applying this equation: minimum sample size = number of items of questionnaire x 5. A total of 240 patients with cardiac surgery were selected from the hospitals with an age range of 40 to 75 years (M=60.24, SD=8.11), of which 97 (40.4%) were females, and 143 (59.6%) were males. Of the participants, 155 (64.6%) had spouses, and 85 (35.4%) were widowed. About the literacy ratio, 102 (42.5%) patients had their education from primary to matric levels, 104 (43.3%) were inter-graduate, and 34 (14.2%) of participants had qualifications from masters to postgraduate level. The employment category showed that 30.0% of participants were in government service, 23.8% worked as private employees, 33.3% had their own business, and 12.9% were retired. Regarding the socioeconomic status of participants, 168 (70.0%) belonged to low economic status, 60 (25%) from the middle class, and 12 (5%) belonged to the higher class

Measures

Brief COPE Inventory

Carver (1997) [16] developed a scale to assess coping skills. The Brief COPE inventory consisted of 26 items and 14 subscales. The scale consisted of two major categories: emotion-focused strategies and problem-focused strategies. Items were scored from “one” to “four.” A higher score indicates a strong ability to cope with stress.

WHO Quality of Life Scale

WHOQOL-BREF is a 26-point system developed by WHO (1995) [17] in four areas: physical wellbeing (seven items), psychological health (six items), social connection (three items), and environmental safety (eight items). The element of the WHOQOL-BREF is graded at the 5-point ordinal scale from 1 to 5, and each element is registered. The findings were then transformed to a 0-100-scale linearly. Mobility, everyday life, productive ability, strength, pain, and sleep are included in physical health. The interventions in the psychological context include a self-image, pessimistic thinking and optimistic attitudes, learning ability, self-esteem, and memory concentration. The social domain consists of personal relations and social support. Issues of financial support, health, living environment, and recreational activities are part of environmental health domains. 

Procedure

After getting institutional review board (IRB) committee approval for the study, the researchers approached the patients during their pre-operative outpatient visit for anesthesia evaluation. The outpatient charge psychiatrist evaluated them for the fulfillment of the criteria. Participants were informed about the design and motives of the study. Guidelines were provided to them in their respective languages, booklets were distributed, and those participants who were not literate were briefed in their native language. Later, participants were followed up during their post-operative days, and the study questionnaire was filled. The confidentiality of the patients was not breached. After collecting the data, questionnaires' scores were calculated, and statistical analyses were drawn out. Results were made while going through statistical analysis. The conclusion was drawn; limitations and suggestions were highlighted to improve the research and attain more beneficial results. A cross-sectional design was selected for conducting this study.

## Results

The results of our study have been described in tables. Table 1 shows that there is a significant correlation found in the subscales of coping strategies and quality of life among patients with cardiac surgery. Coping techniques are positively correlated with quality of life, and there was no significant association of denial with quality of life. While substance use, behavioral disengagement, and self-blame are negatively correlated with physical health, psychological health, social relationship, and the environment.

**Table 1:** Correlation coefficient of coping strategies and quality of life among patients with cardiac disease (N=240) Note: *=p<0.05, **=p<0.01

	Variables	1	2	3	4	5	6	7	8	9	10	11	12	13	14	15	16	17	18
1	Self-Distraction	1	.35**	.02	-.35	.36	.36	-.26	.36*	.42	.37	.16	.32	.42*	.12	.43**	.64**	.34**	.63**
2	Active Coping		1	.32*	.32	.42	.54	.23	19	.03	.15	.43	.32	.35	.24	.75**	.56**	.47**	.42**
3	Denial			1	.34	.21	.31	.23	.23	.42	.24	.23	.24	.52	.23	.13	.04	.05	.16
4	Substance Use				1	.25	.32	.36	.43*	.34	.34	.25	.36	.42	.25	-.53**	-.64*	-.69**	-.63**
5	Use of emotional support					1	.34	.23	.01	.03	.23	.16	.35	.34	.34	.64**	.58**	.43*	.53**
6	Use of instrumental support						1	.32	.15	.35	.36	.34*	.21	.10	.32	.53**	.53**	.46**	.53**
7	Behavior disengagement							1	.32	.24	.34	.24	.21	.01	.05	-.45*	-.69**	-.47**	-.63**
8	Venting								1	.42*	.31	.27	.32	10	.05	.73**	.63**	.53**	.53**
9	Positive reframing									1	.34	.23	.42*	.32	.21	.76**	.68**	.378	.53**
10	Planning										1	.23	.13	.23	.21	.53**	.68**	.73**	.56**
11	Humor											1	.23	.24	.32*	.45**	.58**	.35**	.34**
12	Acceptance												1	.24	.32*	.57	.42	.57**	.63**
13	Religion													1	.29	.53**	.64**	.67**	.43**
14	Self-blame														1	-.64**	-.45*	-.56**	-.33*
15	Physical health															1	.56**	.53**	.46*
16	Psychological Health																1	.47*	.52*
17	Social Relationship																	1	.46*
18	Environment																		1

Table 2 depicts that coping techniques have a significant impact on the QOL of patients with cardiovascular surgery (p<0.05, significance level).

**Table 2:** Linear regression analysis explaining the impact of coping strategies on quality of life (N= 240) Dependent Variable: Quality of Life Note: R²= .012, Adjusted R² =.-.024, F (.593), P<0.05

Model	B	SE	ß	T	P
Constant	43.24	4.01		9.32	.000
Brief COPE	.214	.221	.142	.842	.004

Table 3 displays that there were differences in coping strategies among males and females. Self-distraction was more in males (M=20.9, SD=14.2) as compared to females (M=18.5, SD=.217), t (-4.24), p<0.05. Active coping was seen more in males (M=21.9, SD=.852) as compared to females (M=17.8, SD=.104), t (2.43), p<0.05. Denial was less in males (M=15.2, SD= .392) as compared to females (M=17.5, SD=.390), t (4.56), p<0.05. Use of emotional support was more in males (M=23.5, SD=.452) as compared to females (M=20.4, SD=.615), t (4.42), p<0.05. Behavioral disengagement was more in males (M=24.6, SD=.525) as compared to females (M=29.3, SD=.731), t (3.32), p<0.05. Positive reframing was more in males (M=16.7, SD=.788) as compared to females (M=17.8, SD=.362), t (-4.21), p<0.05. Planning was observed more in males (M=16.1, SD=.232) as compared to females (M=15.4, SD=.268), t (7.20), p<0.05. Acceptance was more in males (M=18.7, SD=.232) as compared to females (M=17.3, SD=.282), t (2.10), p<0.05.

**Table 3:** Mean, standard deviation, t-value, and p-value of each gender on Brief COPE inventory (N= 240) Note: N=number of patients, M=mean, SD=standard deviation, df (degree of freedom)=238, *=p<0.05

Variable	Male: N=130 M(SD)	Female: N=110 M(SD)	t-test	p-value
Self-Distraction	20.9(.142)	18.5(.217)	-4.24	0.00*
Active coping	21.9(.852)	17.8(.104)	2.43	0.00*
Denial	15.5(.392)	17.2(.423)	4.56	0.00*
Substance Use	23.2(.842)	23.6(.642)	-1.43	0.12
Use of emotional support	23.5(.452)	20.4(.615)	4.42	0.00*
Use of Instrumental support	18.3(.342)	17.5(.351)	1.23	0.06
Behavior disengagement	24.6(.525)	29.3(.731)	3.32	0.00*
Venting	17.9(.124)	18.3(.212)	-6.82	0.08
Positive reframing	16.7(.143)	17.8(.362)	-4.21	0.00*
Planning	16.1(.132)	15.4(.268)	-7.20	0.00*
Humor	19.4(.416)	21.9(.342)	-1.21	0.08
Acceptance	18.7(.232)	17.3(.282	-2.10	0.03*
Religion	16.0(.335)	16.8(.423)	-1.21	0.09
Self-blame	13.9(.341)	14.6(.261)	-2.21	0.07

Table 4 shows that there was no significant difference on subscales of QOL and physical health among males (M=22.3, SD=.352) and females (M=20.2, SD=.452), t (-4.60), p>0.05. Psychological health was high in males (M=22.5, SD=.582) as compared to females (M=21.2, SD=.413), t (-1.23), p<0.05. Social relation showed no significant difference among males (M=20.4, SD=.331) as compared to females (M=20.1, SD=.412), t (2.41), p>0.05. There was no significant difference on environment among males (M=20.3, SD= .435) and females (M=21.4, SD=.543), t (-2.53), p>0.05.

**Table 4:** Mean, standard deviation, t-value, and p-value of gender on quality of life scale (N=240) Note: N=number of patients, M=mean, SD=standard deviation, df (degree of freedom)=238, *=p< 0.05

WHOQOL	Male, N=130 M(SD)	Female, N=110 M(SD)	t	P
Physical Health	22.3 (3.52)	20.2(4.52)	-4.60	0.08
Psychological Health	22.5(.582)	21.2(.413)	-1.23	0.04*
Social Relationship	20.4(.331)	20.1(.412)	2.41	.021
Environment	20.3(.435)	21.4(.543)	-2.53	.015

## Discussion

Patients with cardiac surgery experience psychological disturbances, such as anxiety, depression, and stress related to significant mental health issues. This affects the lifestyle quality of individuals. The current study aims to identify patients undergoing cardiac disease and advise them to learn coping strategies for enhancing the quality of life so that one experiences fewer psychological disturbances.

Descriptive statistic of the study shows demographics variables of a person with cardiac disease. The result of Table 1 explains an association between coping and QOL. There was progressive relation among subscales of Brief COPE with different dimensions of QOL, whereas the use of the substance, behavioral detachment, and self-blame is inversely associated with physical, psychological health, social, and environmental relationship. This study also supports the findings that coping techniques predict good QOL, help reduce psychological distress, and improve positive feelings and better functioning [12]. Results suggested a relationship between active coping with high quality of life, whereas avoidant coping is inversely correlated with QOL of the cardiac patients [13]. The findings of the current study are consistent with previous studies indicating that coping is related to QOL.

Table 3 points to the effect of coping on QOL. The findings show that there was a significant impact of coping on the QOL of cardiac surgery patients. Emotion-focused and problem-focused coping strategies lead to a stronger impact on increasing the quality of life while maladaptive techniques decrease it. The findings of the previous studies were also consistent with the present results as most of the patients suffering from disease followed active strategies and were able to cope well with their illness-related stress. Improper ways of coping were not effective for patients while dealing with their problems. Therefore, it is essential to promote positive, healthy, and problem-focused techniques among patients with heart disease, especially those who have a lower quality of life secondary to illness or other comorbidities [14]. Table 4 shows the gender differences in coping strategies used by cardiac patients. There were significant differences in self-distraction active coping, denial, use of emotional support, behavioral disengagement, venting, positive reframing, planning, and acceptance. Research also supports that there is no significant difference in substance use, use of instrumental support, humor, religion, and self-blame [15]. Coping strategies adopted by patients significantly affect their motivation and feelings; hence, they're considered the most important factor in a patient’s overall health and life [16]. Right techniques significantly impact the QOL among cardiac patients. Previous studies point out that problem-focused techniques like acceptance and instrumental support were more effective in enhancing the QOL of patients as compared to emotion-focused techniques such as self-distraction and venting [17,18]. Table 4 findings revealed no significant difference in the quality of life subscales (physical health, social relationship, and environment) among males and females, but a major difference appeared among males and females regarding psychological health. Males scored more on psychological health as compared to females. As the research on gender differences in QOL and coping was not significantly found in previous research, quality of life does not vary according to gender but may vary with other factors [19]. Thus, our findings are consistent with previous research.

Implications and future recommendations

There must be awareness programs to practice coping strategies daily by patients with cardiac surgery. Homework, assignments, and follow-up counseling sessions should be advised to patients to normalize their life, increasing life quality. Further studies should be done with an expanded sample size to ensure external validity. Also, different populations affected by some other diseases should be considered in future research.

## Conclusions

The current study concluded that patients with cardiac heart disease and cardiac surgery are often vulnerable to psychological disturbances and negative thought patterns, which significantly affect their quality of life. If the cardiac patient follows coping strategies, including emotion-focused or problem-focused techniques, it enhances their physical and psychological health and improves interpersonal relationships. These strategies are also helpful in stabilizing financial issues, security, home environment, and daily life functioning.
